# *Molecular Autism*: accelerating and integrating research into neurodevelopmental conditions

**DOI:** 10.1186/2040-2392-1-1

**Published:** 2010-02-22

**Authors:** Joseph D Buxbaum, Simon Baron-Cohen

**Affiliations:** 1 Seaver Autism Center for Research and Treatment, Departments of Psychiatry, Neuroscience, and Genetics and Genomic Sciences, Mount Sinai School of Medicine, New York, NY 10029, USA; 2 Autism Research Centre, Department of Psychiatry, University of Cambridge, Cambridge, CB2 8AH, UK

## 

We are delighted to announce the launch of *Molecular Autism *- a new open-access journal published by BioMed Central. Autism and associated conditions are now recognized as major disabilities with major implications for the public purse. A recent economic analysis of the total annual cost of autism in the UK estimated this to be £28 billion, assuming a prevalence of 1% of the population [1]. The social and communication deficits in this spectrum of conditions, as well as the strongly repetitive behaviors and unusual narrow interests, have their basis in neurodevelopmental changes caused, at the most fundamental level, by molecular alterations, some of which have been identified. The scope of *Molecular Autism *reflects this broad perspective, from molecular to cellular to systems to neural, cognitive and behavioral level analyses, and back again [2].

*Molecular Autism *joins other journals targeting autism and associated conditions but differs in key ways. First, *Molecular Autism *is both online and open access. In a field where dissemination of accurate information has become so important, where internationally, scientists, educators, clinicians, and families are tracking the developments and are looking for up-to-date, definitive and accessible sources of scientific information, *Molecular Autism *will provide such a forum. Open access means that the articles are freely available to all, worldwide, and at no cost to the reader. Online publication also allows for no restriction on number or length of articles and for the inclusion of all available digital technologies: large data sets, unlimited use of color, slide shows, animation, video clips, and links to other web pages, all at no additional charge. Articles are published online on the day of acceptance and, very soon after, are listed in bibliographic databases and full-text article repositories [3]. BioMed Central is a leader in online, open access journals ensuring that this mission will be seamless.

Publication costs in subscription journals are paid by the reader; in *Molecular Autism *these costs are defrayed by the author via article processing charges (APCs). However, APCs can typically be covered by grants. In addition, individual APCs are waived in institutions that have full membership to BioMed Central. A discounted APC is provided for institutions with supporting membership and authors from countries in low or low-middle income categories defined by the World Bank may also have APC charges waived. Beyond this, we will also consider individual waivers on a case-by-case basis, making every effort to ensure that lack of funds does not impede the overall objective of publishing the best science, irrespective of authorship or country of origin [4].

The second way in which *Molecular Autism *differs from other journals in the autism science field is by having a focus on the molecular level. Whereas other journals have a broad remit, from clinical and educational psychological studies through cognitive experiments to neuroimaging and genetics, *Molecular Autism *will publish articles that have a bearing on the molecular basis of autism and related conditions. This does not preclude a behavioural or cognitive or neural study, so long as there is also a molecular measure in the study or the implications for the molecular basis of autism are considered. Our reason for this requirement is that part of our mission statement is to accelerate research into the fundamental determinants of the condition [2]. The first 60 years of autism research have had a bias towards the psychological, and we intend - via this new journal - to correct this emphasis.

The third way in which *Molecular Autism *differs from other autism journals is that it aims to achieve integration across the multiple levels that these syndromes affect. We now recognize that autism will not be understood by a single discipline, be it genetics or neurology or psychology. Rather, each level has to be mapped on to the next, to reveal how mechanisms give rise to the phenotype within each system. This makes autism and related syndromes complex but the response to such a challenge has to be scientific articles that attempt to tackle such multi-level complexity.

We are committed to publishing the highest quality articles in the field. Since we made our first call for manuscripts, we have attracted manuscripts from leaders in the field and have sustained a constant rate of submissions. Articles in *Molecular Autism *are peer-reviewed by at least two, and most typically more, experts, drawn from the Editorial Board and the wider scientific community. Manuscript review and processing is rapid while, with the assistance of the Editorial Board, we shall ensure that the highest standards are maintained, including fair refereeing and editorial decision-making [5].

*Molecular Autism *is also a forum for timely reviews of topics of interest and will be a site for discussion of open questions in the field. After the launch of the journal, we will review the state-of-the-art in association studies in psychiatric genetics and define the criteria for the highest quality studies, and will commit to this level of rigor in all articles published in *Molecular Autism*.

We are in a transformative period in autism research. Behavioral interventions have been shown to be effective, and earlier diagnosis, using molecular and behavioral methods, becomes critical to take advantage of these interventions. In parallel, identification of etiological causes of autism has led to novel drug targets, and several large-scale clinical trials are being carried out based on these findings. We underline the important ethical issue that research into efficacy of medications should evaluate not only improvements in areas of known disability, but the lack of unwanted side-effects (particularly ensuring that areas of strength in autism are not compromised). *Molecular Autism *will be at the forefront of these breakthroughs.
